# Supplementary material for: Investigating supply chain challenges of public sector agriculture development projects in Bangladesh: An application of modified Delphi-BWM-ISM approach
Source: PLoS One. 2022 Jun 22;17(6):e0270254. doi: 10.1371/journal.pone.0270254 (PMC9216582; doi:10.1371/journal.pone.0270254)
Supplement: S3 Table — (DOCX) [file pone.0270254.s003.docx]

**S3 Table. Respondents participated in the ISM brainstorming session**

| **Sl. No** | **Position (level)** | **Years of experience** |
| --- | --- | --- |
| 01 | Executive | 7 years |
| 02 | Assistant Project Manager | 7 Years |
| 03 | Assistant Project Manager | 7 Years |
| 04 | Executive | 8 years |
| 05 | Assistant Project Manager | 8 Years |
| 06 | Executive | 8 Years |
| 07 | Programme Manager | 9 Years |
| 08 | Assistant Project Manager | 10 Years |
| 09 | Procurement consultant | 20 years |
| 10 | Procurement consultant | 20 years |
