# Supplementary material for: Investigating supply chain challenges of public sector agriculture development projects in Bangladesh: An application of modified Delphi-BWM-ISM approach
Source: PLoS One. 2022 Jun 22;17(6):e0270254. doi: 10.1371/journal.pone.0270254 (PMC9216582; doi:10.1371/journal.pone.0270254)
Supplement: S4 Table — (DOCX) [file pone.0270254.s004.docx]

**S4 Table. Average weights of the SCCs of GoB funded projects obtained from BWM, and weights during sensitivity analysis.**

(Global weights for sub-challenges according to sensitivity analysis when the weight of Project/Procurement management issues (A^SCG^) is varied from 0.1 to 0.9)

| **Sub-categories of challenges** |  | **Values of preference weights for listed challenges** | | | | | | | | |
| --- | --- | --- | --- | --- | --- | --- | --- | --- | --- | --- |
|  | **Normal (0.3598)** | **0.1** | **0.2** | **0.3** | **0.4** | **0.5** | **0.6** | **0.7** | **0.8** | **0.9** |
| (A^SCG^_1_) Improper procurement planning | 0.2343 | 0.0651 | 0.1302 | 0.1954 | 0.2605 | 0.3256 | 0.3907 | 0.4558 | 0.5209 | 0.5861 |
| (A^SCG^_2_) Delay in project initiation | 0.1255 | 0.0349 | 0.0698 | 0.1046 | 0.1395 | 0.1744 | 0.2093 | 0.2442 | 0.2791 | 0.3139 |
| (B^SCG^_1_) Demand forecasting error | 0.1142 | 0.1606 | 0.1427 | 0.1249 | 0.1070 | 0.0892 | 0.0714 | 0.0535 | 0.0357 | 0.0178 |
| (B^SCG^_2_) Lack of contract monitoring mechanism | 0.1088 | 0.1529 | 0.1359 | 0.1189 | 0.1019 | 0.0849 | 0.0680 | 0.0510 | 0.0340 | 0.0170 |
| (B^SCG^_3_) Lack of Logistical support | 0.0365 | 0.0513 | 0.0456 | 0.0399 | 0.0342 | 0.0285 | 0.0228 | 0.0171 | 0.0114 | 0.0057 |
| (C^SCG^_1_) Political influence/instability | 0.1120 | 0.1575 | 0.1400 | 0.1225 | 0.1050 | 0.0875 | 0.0700 | 0.0525 | 0.0350 | 0.0175 |
| (C^SCG^_2_) Natural disasters & weather change | 0.0661 | 0.0929 | 0.0826 | 0.0723 | 0.0620 | 0.0516 | 0.0413 | 0.0310 | 0.0207 | 0.0103 |
| (C^SCG^_3_) Biological- disease & pest | 0.0156 | 0.0220 | 0.0195 | 0.0171 | 0.0146 | 0.0122 | 0.0098 | 0.0073 | 0.0049 | 0.0024 |
| (D^SCG^_1_) Lack of competent staff | 0.1155 | 0.1624 | 0.1443 | 0.1263 | 0.1082 | 0.0902 | 0.0722 | 0.0541 | 0.0361 | 0.0180 |
| (D^SCG^_2_) Govt. bureaucracy | 0.0464 | 0.0652 | 0.0580 | 0.0507 | 0.0435 | 0.0362 | 0.0290 | 0.0217 | 0.0145 | 0.0072 |
| (D^SCG^_3_) Lack of institutional ethics | 0.0251 | 0.0353 | 0.0314 | 0.0275 | 0.0236 | 0.0196 | 0.0157 | 0.0118 | 0.0079 | 0.0039 |
| Total | 1.0000 | 1.0000 | 1.0000 | 1.0000 | 1.0000 | 1.0000 | 1.0000 | 1.0000 | 1.0000 | 1.0000 |
