# Supplementary material for: Investigating supply chain challenges of public sector agriculture development projects in Bangladesh: An application of modified Delphi-BWM-ISM approach
Source: PLoS One. 2022 Jun 22;17(6):e0270254. doi: 10.1371/journal.pone.0270254 (PMC9216582; doi:10.1371/journal.pone.0270254)
Supplement: S5 Table — (DOCX) [file pone.0270254.s005.docx]

**S5 Table. Structured Self Interaction Matrix**

| **SCCs** | $D_{3}^{SCC}$ | $D_{2}^{SCC}$ | $D_{1}^{SCC}$ | $C_{3}^{SCC}$ | $C_{2}^{SCC}$ | $C_{1}^{SCC}$ | $B_{3}^{SCC}$ | $B_{2}^{SCC}$ | $B_{1}^{SCC}$ | $A_{2}^{SCC}$ | $A_{1}^{SCC}$ |
| --- | --- | --- | --- | --- | --- | --- | --- | --- | --- | --- | --- |
| $A_{1}^{SCC}$ | A | A | A | V | O | A | V | V | V | A |  |
| $A_{2}^{SCC}$ | A | A | X | V | A | A | X | V | V |  |  |
| $B_{1}^{SCC}$ | A | A | X | V | O | A | V | V |  |  |  |
| $B_{2}^{SCC}$ | A | A | A | V | A | A | A |  |  |  |  |
| $B_{3}^{SCC}$ | A | A | A | V | O | A |  |  |  |  |  |
| $C_{1}^{SCC}$ | V | V | V | O | O |  |  |  |  |  |  |
| $C_{2}^{SCC}$ | O | O | O | V |  |  |  |  |  |  |  |
| $C_{3}^{SCC}$ | A | A | A |  |  |  |  |  |  |  |  |
| $D_{1}^{SCC}$ | X | A |  |  |  |  |  |  |  |  |  |
| $D_{2}^{SCC}$ | X |  |  |  |  |  |  |  |  |  |  |
| $D_{3}^{SCC}$ |  |  |  |  |  |  |  |  |  |  |  |
