# Supplementary material for: Investigating supply chain challenges of public sector agriculture development projects in Bangladesh: An application of modified Delphi-BWM-ISM approach
Source: PLoS One. 2022 Jun 22;17(6):e0270254. doi: 10.1371/journal.pone.0270254 (PMC9216582; doi:10.1371/journal.pone.0270254)
Supplement: S6 Table — (DOCX) [file pone.0270254.s006.docx]

**S6 Table. Final Reachability Matrix with Transitivity Links followed by the modified process**

| **SCCs** | $D_{3}^{SCC}$ | $D_{2}^{SCC}$ | $D_{1}^{SCC}$ | $C_{3}^{SCC}$ | $C_{2}^{SCC}$ | $C_{1}^{SCC}$ | $B_{3}^{SCC}$ | $B_{2}^{SCC}$ | $B_{1}^{SCC}$ | $A_{2}^{SCC}$ | $A_{1}^{SCC}$ | **Driver Power** |
| --- | --- | --- | --- | --- | --- | --- | --- | --- | --- | --- | --- | --- |
| $A_{1}^{SCC}$ | 0 | 0 | 1* | 1 | 0 | 0 | 1 | 1 | 1 | 1* | 1 | **7** |
| $A_{2}^{SCC}$ | 1* | 0 | 1 | 1 | 0 | 0 | 1 | 1 | 1 | 1 | 1 | **8** |
| $B_{1}^{SCC}$ | 1* | 0 | 1 | 1 | 0 | 0 | 1 | 1 | 1 | 1* | 1* | **8** |
| $B_{2}^{SCC}$ | 0 | 0 | 0 | 1 | 0 | 0 | 0 | 1 | 0 | 0 | 0 | **2** |
| $B_{3}^{SCC}$ | 0 | 0 | 1* | 1 | 0 | 0 | 1 | 1 | 1* | 1 | 1* | **7** |
| $C_{1}^{SCC}$ | 1 | 1 | 1 | 1* | 0 | 1 | 1 | 1 | 1 | 1 | 1 | **10** |
| $C_{2}^{SCC}$ | 0 | 0 | 1* | 1 | 1 | 0 | 1* | 1 | 1* | 1 | 1* | **8** |
| $C_{3}^{SCC}$ | 0 | 0 | 0 | 1 | 0 | 0 | 0 | 0 | 0 | 0 | 0 | **1** |
| $D_{1}^{SCC}$ | 1 | 1* | 1 | 1 | 0 | 0 | 1 | 1 | 1 | 1 | 1 | **9** |
| $D_{2}^{SCC}$ | 1 | 1 | 1 | 1 | 0 | 0 | 1 | 1 | 1 | 1 | 1 | **9** |
| $D_{3}^{SCC}$ | 1 | 1 | 1 | 1 | 0 | 0 | 1 | 1 | 1 | 1 | 1 | **9** |
| **Dependence Power:** | **6** | **4** | **9** | **11** | **1** | **1** | **9** | **10** | **9** | **9** | **9** | **78/78** |

1*= Transitive comparison
