# Supplementary material for: Investigating supply chain challenges of public sector agriculture development projects in Bangladesh: An application of modified Delphi-BWM-ISM approach
Source: PLoS One. 2022 Jun 22;17(6):e0270254. doi: 10.1371/journal.pone.0270254 (PMC9216582; doi:10.1371/journal.pone.0270254)
Supplement: S7 Table — (DOCX) [file pone.0270254.s007.docx]

**S7 Table. Level Partition- (all iterations)**

| **SCCs** | **ISM code** | **Reachability Set** | **Antecedent Set** | **Intersection Set** | **Level** |
| --- | --- | --- | --- | --- | --- |
| $A_{1}^{SCC}$ | 1 | 1,2,3,4,5,8,9 | 1,2,3,5,6,7,9,10,11 | 1,2,3,5,9 | III |
| $A_{2}^{SCC}$ | 2 | 1,2,3,4,5,8,9,11 | 1,2,3,5,6,7,9,10,11 | 1,2,3,5,9,11 | III |
| $B_{1}^{SCC}$ | 3 | 1,2,3,4,5,8,9,11 | 1,2,3,5,6,7,9,10,11 | 1,2,3,5,9,11 | III |
| $B_{2}^{SCC}$ | 4 | 4,8 | 1,2,3,4,5,6,7,9,10,11 | 4 | II |
| $B_{3}^{SCC}$ | 5 | 1,2,3,4,5,8,9 | 1,2,3,5,6,7,9, 10,11 | 1,2,3,5,9 | III |
| $C_{1}^{SCC}$ | 6 | 1,2,3,4,5,6,8,9,10,11 | 6 | 6 | V |
| $C_{2}^{SCC}$ | 7 | 1,2,3,4,5,7,8,9 | 7 | 7 | IV |
| $C_{3}^{SCC}$ | 8 | 8 | 1,2,3,4,5,6,7,8,9,10,11 | 8 | I |
| $D_{1}^{SCC}$ | 9 | 1,2,3,4,5,8,9,10,11 | 1,2,3,5,6,7,9,10,11 | 1,2,3,5,9,10,11 | III |
| $D_{2}^{SCC}$ | 10 | 1,2,3,4,5,8,9,10,11 | 6,9,10,11 | 9,10,11 | IV |
| $D_{3}^{SCC}$ | 11 | 1,2,3,4,5,8,9,10,11 | 2,3,6,9,10,11 | 2,3,9,10,11 | IV |
